# Effect of Beetroot Juice Supplementation on Aerobic Response during Swimming

**DOI:** 10.3390/nu6020605

**Published:** 2014-01-29

**Authors:** Marco Pinna, Silvana Roberto, Raffaele Milia, Elisabetta Marongiu, Sergio Olla, Andrea Loi, Gian Mario Migliaccio, Johnny Padulo, Carmine Orlandi, Filippo Tocco, Alberto Concu, Antonio Crisafulli

**Affiliations:** 1 The Department of Medical Sciences, Sports Physiology Lab, University of Cagliari, Via Porcell 4, 09124 Cagliari, Italy; E-Mails: gruppoinforma.marco@tiscali.it (M.P.); silvy_rob@yahoo.it (S.R.); miliaraffaele@gmail.com (R.M.); elisamar84@gmail.com (E.M.); see.olla@tiscali.it (S.O.); filippo.tocco@tiscali.it (F.T.); concu@unica.it (A.C.); 2 Regional School of Sport of Sardinia, Italian Olympic Committee, Cagliari, Italy; E-Mails: loindr67@gmail.com (A.L.); ciao@migliaccio.it (G.M.M.); 3 Tunisian Research Laboratory “Sports Performance Optimisation” National Centre of Medicine and Science in Sports (CNMSS), Tunis, Tunisia; E-Mail: acrisaful@virgilio.it; 4 Faculty of Medicine and Surgery, University of Tor Vergata Rome, Rome, Italy; E-Mail: crisaful@unica.it

**Keywords:** exercise, nitric oxide, aerobic energy cost, anaerobic threshold, oxygen uptak

## Abstract

The beneficial effects of beetroot juice supplementation (BJS) have been tested during cycling, walking, and running. The purpose of the present study was to investigate whether BJS can also improve performance in swimmers. Fourteen moderately trained male master swimmers were recruited and underwent two incremental swimming tests randomly assigned in a pool during which workload, oxygen uptake (VO_2_), carbon dioxide production (VCO_2_), pulmonary ventilation (VE), and aerobic energy cost (AEC) of swimming were measured. One was a control swimming test (CSW) and the other a swimming test after six days of BJS (0.5l/day organic beetroot juice containing about 5.5 mmol of NO_3_^−^). Results show that workload at anaerobic threshold was significantly increased by BJS as compared to the CSW test (6.3 ± 1 and 6.7 ± 1.1 kg during the CSW and the BJS test respectively). Moreover, AEC was significantly reduced during the BJS test (1.9 ± 0.5 during the SW test *vs.* 1.7 ± 0.3 kcal·kg^−1^·h^−1^ during the BJS test). The other variables lacked a statistically significant effect with BJS. The present investigation provides evidence that BJS positively affects performance of swimmers as it reduces the AEC and increases the workload at anaerobic threshold.

## 1. Introduction

A diet rich in vegetables has been found to have a beneficial impact on several body functions [1]. These effects may, in part, be attributable to the high inorganic nitrate (NO_3_^−^) content of vegetables. NO_3_^−^ can be reduced to nitrite (NO_2_^−^) and in turn to nitric oxide (NO), which affects hemodynamics and muscle metabolic functions [2,3]. In this regard, recent evidence suggests that beetroot juice supplementation may positively impact the physiological responses to exercise. In detail, it has been found that beetroot juice can enhance NO production in the skeletal muscle, thereby increasing blood flow and improving muscle O_2_ delivery [2,4]. Moreover, a reduction in O_2_ cost of exercise has been demonstrated as well as improved performance, even though the precise mechanism through which dietary nitrate supplementation operates is not yet fully understood [4,5,6]. It appears that these effects are related to NO_2_^−^ and NO-mediated enhancements of muscle contractile function, and/or mitochondrial efficiency and/or enhanced muscle blood flow [4,5,6,7,8,9,10]. The mechanisms by which nitrates act to enhance exercise efficiency remain speculative. Is is suggested that increased levels of nitric oxide may reduce the ATP cost of force production through a regulation of the ATP consuming processes or myofibrillar actin-myosin interaction in force production. Moreover, there is evidence that elevations of NO improves muscle metabolism, thereby modulating the ATP cost of force production [9,10].

The beneficial effects of beetroot juice supplementation (BJS) have been tested in cycling, walking, and running [3,6]. However, to the best of our knowledge no studies have been conducted investigating the effect of BJS in swimming. This is probably because the measurement of certain physiological parameters such as oxygen uptake (VO_2_) and anaerobic threshold (AT) during swimming is hampered by technical limitations due to the difficulty of assessment of expired gases in a pool. Our group has recently developed a method to measure gas exchange during incremental tethered swimming by using an adapted snorkel that is able to gather respiratory parameters without the need of any cumbersome apparatus such as the Douglas bag [11]. By means of this device, the uniqueness of swimming testing with respect to laboratory procedures for exercise capacity such as cycling, running, and arm cranking has been demonstrated. This investigation has provided evidence that none of the laboratory tests utilised to measure oxygen uptake in swimmers yielded results comparable to those obtained by tethered swimming testing [11]. This fact has strengthened the concept that swimmers should be tested in their specific activity. Moreover, the most specific parameter appeared to be the anaerobic threshold (AT), which showed the most relevant difference between swimming and the other tests. Thus, to demonstrate any beneficial effect of food supplementation in swimmers a specific swimming test should be employed. The importance of dietary supplementation able to improve aerobic metabolism is of particular interest in swimming, where there is the predominant influence of the aerobic system during the performance.

The purpose of this investigation was to ascertain whether performance in swimmers could be improved by a week of beetroot juice supplementation. Expiratory gas exchange was collected together with workload imposed during an incremental tethered swimming test in a pool. This allowed for the calculation of aerobic cost of exercise to test the hypothesis that, as similarly demonstrated for walking, running, and cycling, beetroot juice supplementation can reduce the O_2_ cost of swimming.

## 2. Methods

### 2.1. Subjects

Fourteen moderately trained male swimmers were recruited to take part in this investigation. The level of fitness was judged moderate on the basis of their maximum oxygen uptake (VO_2max_) evaluated by means of a standard incremental exercise test on a cycle-ergometer. Their mean ± standard deviation (SD) of age, mass, and height were 34.7 ± 7.5 years, 69.4 ± 6.1 kg, and 173.6 ± 4.3 cm respectively, while VO_2max_ was 42.7 ± 2.6 mL∙kg∙min^−1^. All subjects were master athletes who were regularly involved in regional and national competitions and who trained an average of 6.5 ± 0.8 h per week. The training frequency ranged from 3 to 4 times/week, with 3.000–5.000 m distance covered each time. All were in the middle phase of the training season. None had any history of cardiac or respiratory disease or was taking any medication at the time of the study and none showed any abnormalities on physical examination or on resting electrocardiogram. Written informed consent was obtained from all of the participants after they were informed about the methods and aims of the study. The study protocol was approved by the ethical committee of the University of Cagliari and carried out according to the Declaration of Helsinki.

### 2.2. Experimental Design

**Figure 1:**
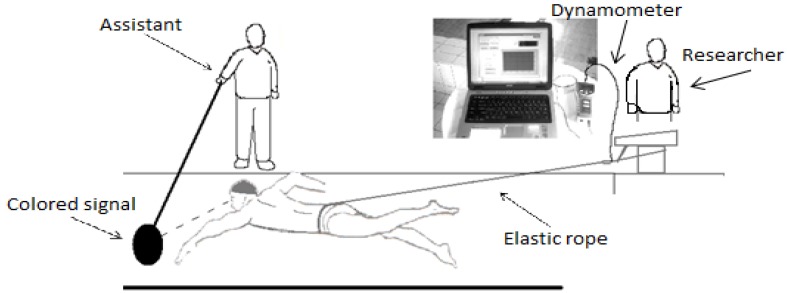
Schematic representation of the tethered-swimming apparatus. By means of a dynamometer, the tension applied to the elastic rope was constantly monitored on the display. Continuous vocal feedback was provided by the researcher who checked the dynamometer to an assistant who moved a pole with a coloured signal fixed at the extremity and immersed in the water forward or backward. The tested swimmer was instructed to follow the signal so that the assistant could adjust the tension applied to the rope simply by moving the pole forward or backward.

Each participant underwent the following protocol randomly assigned to eliminate any order effect:

- Incremental control swimming (CSW) test: this test consisted of tethered swimming, conducted between 10 a.m. and 2 p.m. in a 25-metre indoor swimming pool, under the same water condition (water temperature of 27 °C). All swimmers performed front crawl with free stroke frequency. The athlete was attached by a waist belt to an elastic rope connected to a digital dynamometer (PCE, FM1000, Ballingen, Germany) able to continuously provide on a display the values of the force being applied to the rope. The belt arrangement allowed the swimmers to kick freely. The other extremity of the dynamometer, which had a computer interface which allowed for the registration of the tension applied to the rope throughout tests, was fixed to the starting block of the pool. The instrument range of measure was from 0 to 100 kg, with a resolution of 0.05 kg and an accuracy s = 0.5% (±5 N). The tension applied to the elastic rope was constantly monitored on the dynamometer display, and the researcher (who checked the dynamometer) provided continuous vocal feedback to an assistant who moved a pole with a coloured signal fixed at the extremity and immersed in the water forward or backward. The tested swimmer was instructed to follow the coloured signal so that the assistant could adjust the tension applied to the rope simply by moving the pole forward or backward (Figure 1). Through use of a previously described method [11], an incremental test was developed, which started from a workload of three kg and was increased progressively by one kg.min^−1^. The test terminated when the athlete was no longer able to maintain his position and to follow the coloured signal for more than 30 s.
- Incremental swimming after beetroot juice supplementation (BJS) test: this was conducted in the same manner as the CSW test, but athletes performed the test after a week of beetroot juice supplementation. In detail, athletes received 6 days of dietary supplementation with NO_3_^−^ rich beetroot juice (Reed Beet Juice, Aureli, Ortucchio, Italy). In detail, they received 0.5l/day organic beetroot juice containing about 5.5 mmol of NO_3_^−^ [6].

Subjects consumed a light meal at least two hours (interval 2–5 h) before testing. Meal consisted in a typical Italian breakfast with toasted bread, biscuits, fruit jam, honey, and milk. Subjects could eat *ab libitum.* Subjects were also asked to avoid caffeine and alcohol the day before tests were scheduled.

Throughout the CSW and BJS tests participants’ expired gases were analysed by a portable metabolic device (VO2000, MedicGraphics, St. Paul, MN, USA), which provided a 3-breath average of VO_2_, VCO_2_, and pulmonary ventilation (VE) through telemetric transmission. This system has been shown to be reliable and to have a good agreement compared to a standard metabolic cart for laboratory use [12,13]. Moreover, the VO2000 was able to gather HR values from a chest belt connected to the VO2000 by telemetric transmission. During both tests athletes wore a face mask and a breathing valve connected to the VO2000 for expired gases analysis. This system was modified for tethered swimming. In this setting, athletes breathed through a low volume (about 200 mL) corrugated flexible plastic tube attached to the VO2000 breathing valve. This system had already been used in a similar experiment session to collect gas exchange in a swimming pool [11].

Tests were set apart by at least two weeks (range 2–4 weeks) and athletes were asked to have a meal at least three hours before testing.

During tests the following parameters were measured at both anaerobic threshold (AT), which was determined using the V-slope method, and at maximum workload achieved: workload, VO_2_, VCO_2_, VE, HR, and aerobic energy cost [14]. In particular, the aerobic energy cost of swimming (AEC, kcal·kg^−1^·h^−1^) was calculated by dividing the average oxygen uptake (indexed by body mass) every minute by the tension applied to the rope (in kg) and multiplying this quantity by 60. By utilising the Weir equation, the resulting quantity was further multiplied using the following equation:

AEC= 3.941·VO_2_ + 1.106·VCO_2_ to obtain kcal from oxygen uptake [15]. This equation was used when the respiratory exchange ratio (RER) was <1, while an oxygen caloric equivalent of 5.04 was used when RER became >1. In this case, it was assumed that all aerobic energy was derived from carbohydrate oxidation. Maximum oxygen uptake (VO_2_max) was considered as the attainment of at least two of the following criteria: (1) a plateau in VO_2_ despite increasing speed (<80 mL∙min^−1^); (2) a respiratory exchange ratio (RER) above 1.10; and (3) a heart rate (HR) ± 10 beats∙min^−1^ of predicted maximum HR calculated as 220-age [16]. Calculation of VO_2_max was carried out during the last 30s of the last exercise step.

Prior to testing, the VO2000 was calibrated according to the manufacturer’s instructions. For the calibration, the additional dead space of the plastic tube was taken into account by modifying the pre-set parameters of the VO2000.

*Calculation and data analysis.* Results are presented as mean ± SD. The *t t*est for paired data was performed in order to compare data between tests CSW and BJS. Significance was set at a *p* value of <0.05. Descriptive statistics were carried out before the *t* test to confirm the assumptions of normality by means of the Kolmogorov-Smirnov test. The alpha level was set at *p* < 0.05. Statistics were calculated employing commercially available software (Graph-Pad Prism).

## 3. Results

**Figure 2:**
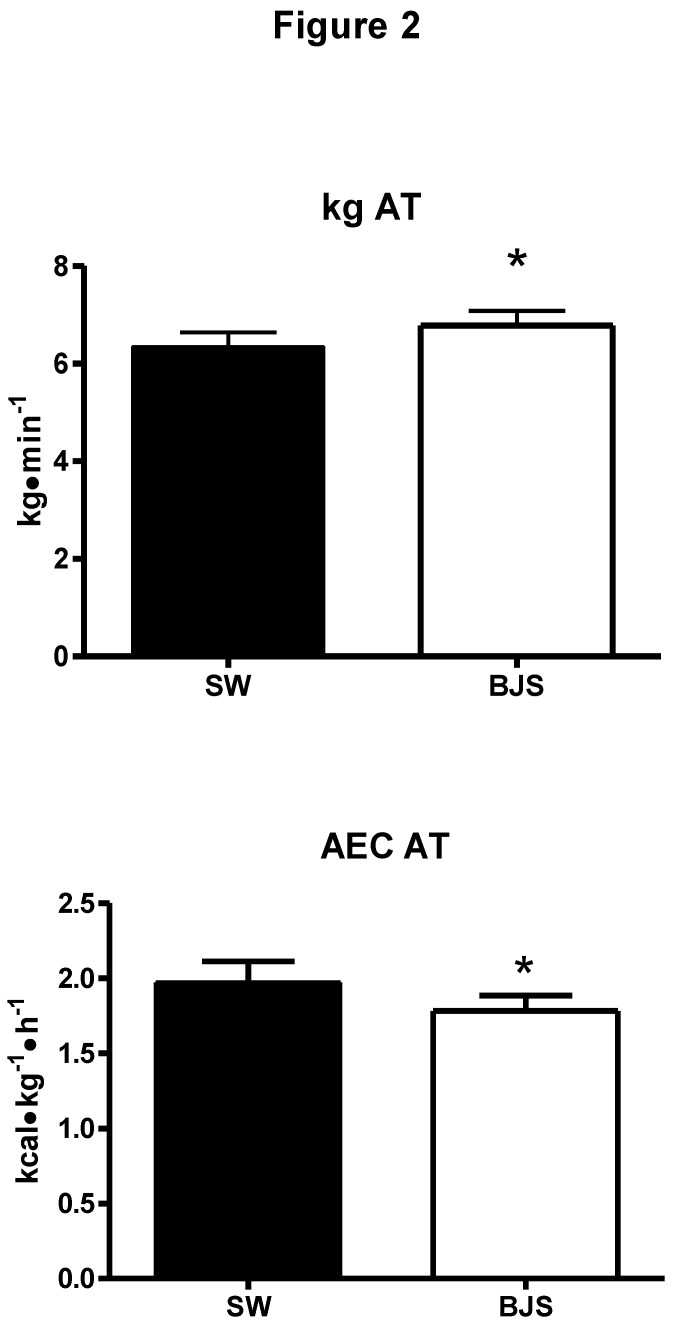
Upper panel: workload at anaerobic threshold (kg AT panel: aerobic energy cost at anaerobic threshold (AEC AT). CSW = control swimming test; BJS = beetroot juice supplementation test. * = *p* < 0.05 *vs.* the CSW test.

Table 1 shows HR, VO_2_, VCO_2_, and VE values at rest before CSW and BJS tests. Statistics did not highlight any difference between the two settings, thus athletes started tests from similar conditions. Figure 2 shows parameter values which reached statistical significance. In particular, the upper panel demonstrates that workload at AT was significantly increased by beetroot juice supplementation as compared to the CSW test. In detail, workload reached at AT was 6.3 ± 1.0 and 6.7 ± 1.1 kg during the CSW and the BJS test respectively. The lower panel of Figure 2 also demonstrates that AEC was significantly reduced during the BJS test, as this parameter reached a level of 1.9 ± 0.5 during the CSW test and 1.7 ± 0.3 kcal·kg^−1^·h^−1^ during the BJS test. None of the other variables measured at anaerobic threshold (*i.e.*, VO_2_, VCO_2_, VE, and HR) were significantly affected by beetroot juice supplementation, as shown by Table 2. The kg level at maximum workload was effected but not at a significant value. It is likely that increasing the number of participants would have resulted in a statistically significant outcome.

**Table 1:** baseline level in oxygen uptake (VO_2_), carbon dioxide production (VCO_2_), pulmonary ventilation (VE) and heart rate (HR) at rest before control swimming (CSW) and beetroot juice supplementation (BJS) tests.

	*CSW*	*BJS*	*p* value
VO_2_ (mL min^−1^)	677 ± 228	548 ± 213	0.134
VCO_2_ (mL min^−1^)	769 ± 251	757 ± 312	0.911
VE (L min^−1^)	33.10 ± 11.3	33.25 ± 14.2	0.975
HR (bpm)	59 ± 8	57 ± 9.5	0.552

**Table 2:** variables’ level reached at anaerobic threshold (AT) and maximum workload (MAX) during the control swimming (CSW) and the beetroot juice supplementation test (BJS). Kg = workload, VO_2_ = oxygen uptake, VCO_2_ = carbon dioxide production, VE = pulmonary ventilation, HR = heart rate, aerobic energy cost = AEC.

	AT			MAX		
	*CSW*	*BJS*	*p* value	*CSW*	*BJS*	*p* value
kg (kg∙min^−1^)	6.35 ± 1	6.7 ± 1.1	0.008	7.7 ± 1.4	8 ± 1.5	0.101
VO_2_ (mL∙min^−1^)	2817 ± 545	2741 ± 454	0.481	2989 ± 530	3066 ± 635	0.384
VCO_2_ (mL∙min^−1^)	2678 ± 453	2732 ± 402	0.662	3361 ± 694	3381 ± 663	0.864
VE (L∙min^−1^)	75.7 ± 14.2	77.7 ± 19.4	0.574	96 ± 19.6	99.5 ± 30.9	0.415
HR (bpm)	152 ± 12	155 ± 13	0.548	183 ± 8	186 ± 6	0.301
AEC (kcal∙kg^−1^∙h^−1^)	1.9 ± 0.5	1.7 ± 0.3	0.040	1.7 ± 0.4	1.6 ± 0.3	0.527

## 4. Discussion

The purpose of the present study was to ascertain whether performance in swimmers could be improved by a week of beetroot juice supplementation. Results show that beetroot juice supplementation reduced aerobic energy cost of swimming at submaximal workload, as shown by the reduced AEC at anaerobic threshold found in the present investigation. This finding is in agreement with previous research showing that dietary NO_3_^−^ supplementation by beetroot juice can reduce aerobic cost of submaximal strain in various kinds of exercise [3,5,6,17,18]. To the best of our knowledge this is the first study to investigate the effects of BJS in swimming.

The precise manner by which dietary NO_3_^−^ supplementation can affect aerobic efficiency at sub-maximal workloads remains unclear, but several mechanisms have been postulated to explain this phenomenon. In detail, a NO-mediated enhancement in mitochondrial efficiency and/or enhanced muscle blood flow have been reported, even though some have argued that this effect could be related to NO-mediated effect on muscle contractile function [2,4,6,7,8,17].

Therapeutic nitrates have traditionally been used for their capacity to dilate vessels in ischemic cardiovascular disease. In addition, nitrates have been found to improve the efficiency (*i.e.*, they can reduce energy cost) of exercise as indicated by a 4%–5% reduction in oxygen uptake at steady state [5,6,17]. The mechanism by which nitrates act to enhance exercise efficiency remains contentious. Is hypothesized that increased levels of nitric oxide following supplementation may reduce the ATP cost of force production [17]. It has been suggested that the reduction of ATP use is a consequence of nitric oxide’s regulatory effect on the ATP consuming processes of sarcoplasmic reticulum calcium pumping [19] or myofibrillar actin-myosin interaction [20] in force production. Moreover, there is evidence that small elevations of NO improves muscle metabolism, preventing excess calcium release and subsequently modulates the ATP cost of force production [9]. Taken together, these facts support the concept that nitrate supplementation reduces the oxygen cost of exercise through increasing the efficiency of energy production [9].

In the present study, an increase in the workload attained at anaerobic threshold was also found, thereby suggesting that an improvement in the capacity to perform work independently from anaerobic energy sources took place after BJS. This finding suggests that the reduction in O_2_ cost afforded with BJS was also able to increase exercise performance during swimming.

However, it should be noted that no enhancement was found in the maximum workload achieved and/or VO_2max_. This phenomenon is consistent with the concept that the effect of BJS on high intensity exercise may be independent from the effects of BJS on the oxygen cost of submaximal exercise [4]. Likewise, there was no effect on respiratory variables at rest, which is similar to what was recently found by Wylie and co-workers (2013) [4] using various doses of beetroot juice supplementation. These authors reported a slight increase in respiratory exchange ratio, due to an increase in VCO_2_ production, only for high beetroot dosage. They attributed this effect to the sugar content of the beverage.

Results of the present research may have potentially practical applicability as it indicates that BJS may be suitable to enhance exercise performance in swimming. The fact that an increase in performance was detected only at anaerobic threshold deserves attention. It is well known that AT is more sensitive than VO_2max_ in detecting the specificity of training and that AT should be preferentially used when evaluating swimmers’ fitness status [11]. It has been suggested that VO_2max_ is a valuable tool to distinguish between fit and unfit subjects, but it is not sensitive enough to discriminate between subjects of homogeneous performance levels, since it suffers from limitation due to the fact that adequate motivation of the subject is necessary to appropriately determine VO_2max_ [21]. On the other hand, sub-maximal parameters such as AT are less sensitive to motivation [21]. Moreover, while VO_2max_ reflects the integration of several mechanisms (ventilation, cardiac output, peripheral muscle O_2_ extraction etc.), AT is a phenomenon that may depend on several complex mechanisms. Among others, AT may reflect type II (fast-twitch glycolitic) muscle fibres recruitment, which are sensitive to training [22]. In fact, it has been reported that anaerobic and ventilatory thresholds are important variables distinguishing endurance performance in athletes homogeneous in terms of VO_2max_, and that AT is a more useful indicator of aerobic endurance performance than VO_2max_ [21,23,24].

The improved swimming performance at anaerobic threshold found in the present investigation suggests that BJS influences preferentially type 2 muscle fibres. This suggestion is in accordance with recent evidence in rats, where BJS induces a marked increase in muscle blood flow during exercise with the blood flow preferentially distributed to muscle groups that principally contain type 2 fibres, which are recruited especially during moderate-high intensity exercise. Moreover, it was found that BJS results in a preferential distribution of blood flow to type 2 fibres and improves oxidative functions in hypoxic muscle [2,25].

Another fact deserving attention is that the present research was conducted on moderately trained master athletes. Thus, it is not known whether BJS can also confer a performance benefit in elite swimmers. Several clues indicate that performance might not be enhanced to the same extent by BJS in highly trained athletes [26,27]. This may relate to factors such as greater nitric oxide synthase activity, better muscle oxygenation and mitochondrial efficiency, and a lower fraction of type II fibres in the muscles of highly trained, compared with moderately trained, individuals [27].

### 4.1. Limitations of the Study

One possible weak point of the present study is the lack of a placebo group. This fact did not allow the drawing of solid conclusions. Actually, a placebo effect was possible and this is a major limitation. However, BJS improved AEC and workload only at AT, while parameters were unaffected at VO_2max_. As previously stated, while VO_2max_ depends on subjects' motivation, AT are less sensitive to motivation, thus rendering AT less dependent on a possible placebo effect. Further study is warranted to rule out any possible placebo effect of BJS on swimming performance.

## 5. Conclusions

In conclusion, results of the present study provide evidence that beetroot juice supplementation may positively affect performance of trained master swimmers. This beneficial effect results in a reduction of aerobic energy cost and increased workload at anaerobic threshold, whereas maximum oxygen uptake and maximum workload are not affected. Further research is needed to clarify whether beetroot juice supplementation may also be beneficial for highly trained swimmers.
